# Surfactant-free synthesis of copper nanoparticles and gas phase integration in CNT-composite materials†

**DOI:** 10.1039/d0na00922a

**Published:** 2020-12-10

**Authors:** Paul Brunet, Ruairi J. McGlynn, Bruno Alessi, Fiona Smail, Adam Boies, Paul Maguire, Davide Mariotti

**Affiliations:** Nanotechnology and Integrated Bio Engineering Centre (NIBEC), Ulster University Newtownabbey BT370QB UK d.mariotti@ulster.ac.uk; Department of Engineering, Cambridge University Cambridge UK

## Abstract

Copper nanoparticles (Cu-NPs) represent a viable low-cost alternative to replace bulk copper or other more expensive NPs (*e.g.* gold or silver) in various applications such as electronics for electrical contact materials or high conductivity materials. This study deals with the synthesis of well dispersed Cu-NPs by using an Ar + H_2_ microplasma using a solid copper precursor. The morphological analysis is carried out by electron microscopy showing particles with a mean diameter of 8 nm. Crystallinity and chemical analyses were also carried out by X-ray diffraction and X-ray photoelectron spectroscopy, respectively. In the second step, the Cu-NPs were successfully deposited onto porous carbon nanotube ribbons; surface coverage and the penetration depth of the Cu-NPs inside the CNT ribbon structure were investigated as these can be beneficial for a number of applications. The oxidation state of the Cu-NPs was also studied in detail under different conditions.

## Abstract 2

Surfactant free synthesis of copper nanoparticles by using atmospheric plasma pressure.

## Graphical abstract

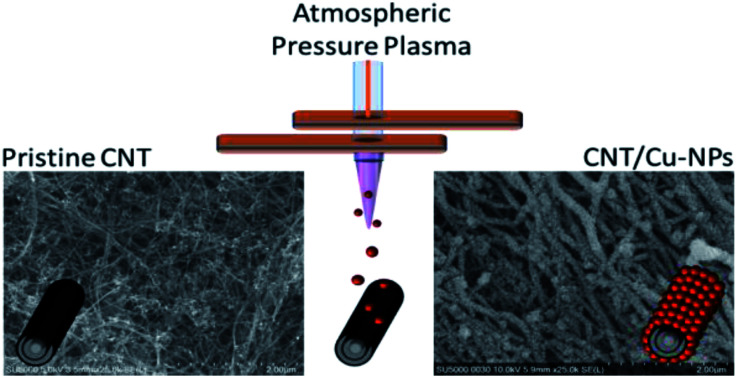

## Introduction

Metallic nanoparticles (NPs) continue to attract a lot of attention as their small size confers them unique properties that have been already exploited in different fields such as medicine,^1,2^ optics^3^ and sensors.^4^ Nowadays the necessity of materials with multifunctional properties urges researchers to combine metallic NPs with other materials such as graphene, cellulose or carbon nanotubes (CNTs).^5–11^

Gold and silver remain the most widely studied elements used in metallic NPs due to their absorption in the visible range, good electrical conductivity and also because of their chemical stability.^12,13^ However, their cost can be prohibitive in various applications where copper represents a viable alternative.

In the literature, different techniques have been investigated to produce copper NPs (Cu-NPs).^14^ As it is the case for most materials, chemical and physical methods are generally popular for the synthesis of Cu-NPs. Chemical methods often rely on chemical compound precursors and stabilizers such as CuCl_2_,^15^ CuSO_4_ (ref. 16) or Cu(NO_3_)_2_,^17^ which in some cases present safety, health and environmental concerns, and synthesis times that can go from hours to days involving generally several steps.^18^

Physical methods based on plasma processes are generally advantageous for NP synthesis because they do not require surfactants/reducing agents and purification steps are therefore eliminated. Several physical methods have been therefore used for producing Cu-NPs.^19,20^

Plasmas operated at atmospheric pressure have been also used for this purpose presenting some benefits compared to other methods. Xu Zhijian *et al.* investigated the use of dielectric barrier discharges fed with hydrogen (H_2_) to reduce copper oxide and obtain Cu-NPs (∼35 nm in diameter).^21^ Masaaki Nagatsu *et al.* investigated an Ar/H_2_ radio-frequency (RF) microplasma with a copper wire sacrificial electrode (1 mm diameter) showing the possibility of producing nanostructured copper thin films.^22^ Another study by G. Dinescu *et al.* was carried out with an argon RF microplasma jet without hydrogen, also with a sacrificial copper electrode (a copper cylinder in this case).^23^ Microplasma-based processes can be considered a generalized approach for the synthesis of a wide range of metal and metal oxide nanoparticles.^24–29^ However, in the specific case of metallic Cu-NPs, a detailed analysis of mean size, size distribution and of the chemical composition (*e.g.* degree of oxidation) of NPs produced by microplasmas is still missing. In particular the degree of oxidation is an important aspect as it can impact opto-electronic properties such as conductivity as well as their chemical stability.^30,31^ Exploring the possibility of utilizing these processes to produce composites is an important aspect that has not been reported widely.

The combination of CNTs and Cu-NPs in a composite material has shown great potential for applications where mechanical, thermal and electronic properties need to be tailored to meet the requirements of *e.g.* the telecommunication or automotive industry.^32^ In order to synthesize CNTs/NP composite materials, NPs are generally deposited on macroscale support structures made of CNTs, which generally exhibit a porous 3D microscale architecture.^33^ The properties of the composite material therefore strongly depend on the loading, surface coverage and on penetration depth of the NPs within the 3D porous structure. For instance, for high conductivity applications, surface coverage appears to play a key role;^34^ however, this is also a major concern for high-conductivity light-weight composites as increasing the NP loading also increases the weight. Surface and interface states of the Cu-NPs (*e.g.* oxidation) with CNTs are also of relevance and the use of surfactants is usually required. However this can alter the properties of the composite.^32^

Here we report a simple method to realize surfactant-free metallic Cu-NPs and CNTs/Cu-NP composite structures. We first present a detailed analysis of the Cu-NP size, size distribution, chemical composition and oxidation state under different conditions. We then produce CNTs/Cu-NP composites and investigate their morphology, CNT surface coverage and evaluate the CuNP penetration depth within the 3D CNT architecture. Our results show the feasibility of CNTs/Cu-NP composites with a two-step process where a high degree of surface coverage is achieved. We also shed light on the ability of the process to insert NPs deep in the CNT porous structure.

## Plasma reactor description and procedures

**Fig. 1:**
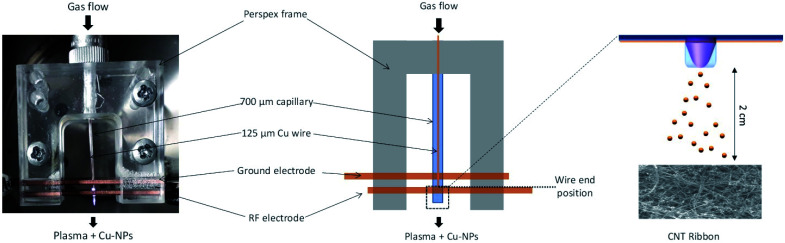
Photograph of the real plasma reactor and the corresponding schematic drawn.

A picture of the real plasma reactor and a schematic drawn are provided in Fig. 1.

## Materials preparation and characterization

Collection or deposition of the NPs was carried out by following different procedures. The NPs were collected directly in ethanol as a colloid or directly onto grids for transmission electron microscopy (TEM) or were deposited on silicon substrates or carbon nanotubes (CNTs) for X-ray diffraction (XRD), scanning electron microscopy (SEM) equipped with an energy dispersive spectroscopy (EDX) and X-ray photoelectron spectroscopy (XPS). Cu-NPs were characterized both independently and as part of CNT/Cu-NP composite samples. For the composite samples, multi-wall CNTs were synthesized following a well-established process (see ref. 35 and 36 and see the ESI S2†) which produces CNT ribbons ∼2 cm wide, 3–7 μm thick and up to 15 cm long; however, here we have used CNT ribbons that were cut into 2 cm long samples (see the ESI S2.2, S2.3†). The CNT ribbons were then placed on silicon substrates and to improve adhesion they were wetted with ethanol at the CNT ribbon ends outside the deposition area and dried for 2 h. In order to produce the composite samples, CNT ribbons on silicon were placed inside the chamber and Cu-NPs were deposited from a distance of ∼2 cm from the end of the capillary tube as shown in Fig. 1. This distance was determined to be the closest distance required to repeatedly avoid any coupling between the plasma and the samples, which would lead to plasma heating and sample damage. Characterization of all samples was carried out immediately after deposition of Cu-NPs.

The Cu-NPs and CNTs/Cu-NP composites were examined by TEM and high-resolution TEM (HR-TEM) using a JEOL JEM-2100F electron microscope operated at 600 keV. Size distribution of the particles was acquired at 200 keV. In the case of the CNTs/Cu-NP composites, the samples were sonicated for a few seconds with an ultrasonic probe (sonics Vibra cell model CV 188) to disperse the CNTs/Cu-NPs in ethanol before drop casting the colloidal suspension on the holey carbon mesh (300)/Au TEM grids and dried for a night. The analysis of the size distribution of the Cu-NPs and the fast Fourier transform of the HRTEM images was carried out with ImageJ software.^37^

Structural properties of the Cu-NPs deposited directly on Si substrates were assessed by XRD and the spectra were acquired with a Bruker D8 Discover using Cu-Kα radiation (*λ* = 1.5418 Å) in Bragg–Brentano geometry (*θ*–2*θ*) in the range of 30–80° with a step of 0.02°.

X-ray photoelectron spectroscopy (XPS) was used in this study with the aim of analyzing the composition and evaluating the purity and the degree of oxidation through the identification of chemical bonds.

Cu-NPs were also in this case deposited directly on Si substrates or onto the CNT ribbons. The spectrometer was an ESCALAB XI^+^ instrument, ThermoFisher.

The pressure in the fast entry lock was higher than 2 × 10^−6^ mbar prior to transfer to the analysis chamber, achieved with an Edwards RV5 rotary vane pump. The base pressure during spectral acquisition was higher than 5 × 10^−7^ mbar achieved by using an Edwards E2M28 rotary vane pump. Residual gas analysis revealed that the main background gas in the analysis chamber was argon. The excitation source was a monochromated aluminium anode with an excitation energy of 1486.7 eV operated at 15 kV and 15 mA giving a source power of 225 W. The recorded spectra include C 1s, O 1s, Cu 2p, Si 2p and Pt 4f (as a reference), which were acquired sequentially with a total acquisition time of 8 min.

With the selected scan parameters, the energy resolution was 0.1 eV for high-resolution spectra and 1 eV for survey spectra. The size of the analyzed sample area was 650 μm and takes the form of an elongated circle. The transfer procedure within the spectrometer includes exposure to 5 × 10^−7^ mbar for 5 min. Charge compensation, by means of an electron beam, was applied *via* a flood gun operated at 100 μA. The charge referencing method was performed by shifting the asymmetric Pt 4f_7/2_ peak of freshly sputter-cleaned Pt foil to 71.2 eV. The foil was pressed onto the surface of the CNT ribbons by using a copper clip and the contact between the sample and the sample bar was confirmed by the Fermi level in both cases remaining close to 0 eV. The sputter cleaning was performed with a monoatomic argon source held at 4000 eV and a current of 1 μA applied over a 1.5 mm wide square.

The surface morphology of the CNTs/Cu-NP samples was investigated by using a SEM Hitachi SU5000.

Images were acquired in the high-resolution mode with an extraction voltage of 10 kV and magnification varying from ×25k to ×120k. Chemical analysis as a function of the thickness was performed using EDX and by varying the voltage from 1 kV to 30 kV.

## Results and discussion

### Characterization of the Cu-NPs

Cu-NPs were successfully synthesized using a RF atmospheric pressure microplasma with an applied power of 60 W (Fig. 1). The use of hydrogen in the plasma gas mixture was necessary for the synthesis to achieve observable deposition on a substrate. This requirement was similar to that for the synthesis of gold NPs and was attributed to the exothermic reaction of the recombination of atomic hydrogen, from H_2_, which enhances metal etching.^38^

**Fig. 2:**
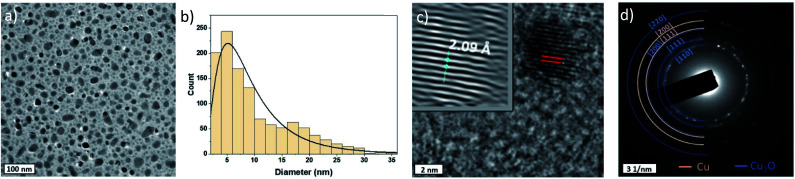
(a) Low resolution TEM image of the as deposited Cu-NPs; (b) size distribution of TEM images; (c) high resolution TEM image and d-spacing of the [111] and [200] planes of Cu and (d) selected area electron diffraction (SAED) pattern of the sample.

Fig. 2a shows a representative low resolution TEM image of the Cu-NPs, which generally present spherical shapes. We have analysed >1000 particles and produced the size distribution (Fig. 2b), which broadly follows a log-normal trend with a geometric mean diameter of 7.94 nm and a geometric standard deviation of 1.92.

Nevertheless, particles as large as 30 nm in diameter were also found, which can be in part attributed to coalescence of smaller particles in flight during synthesis;^39–42^ this is corroborated by a slightly bimodal distribution with a peak developing above ∼16 nm diameter. NPs are well dispersed and non-agglomerated.

The composition of the NPs was determined by measuring the *d*-spacing in HRTEM images on isolated Cu nanoparticles as shown in Fig. 2c, representative of a larger number of particles analysed. First, fast Fourier transforms (FFTs) are applied on the particle images with clear lattice fringes, then a mask is applied and finally an inverse FFT (iFFT) allows obtaining the features in the top left corner of the TEM image in Fig. 2c. The measured *d*-spacing from the profile of the iFFT is found to be 0.209 nm which corresponds to the [111] plane of Cu. Nevertheless, the SAED pattern acquired shows that while some of the rings belong to Cu [111] and [200] planes, others can be assigned to Cu_2_O [110], [111] and [220] planes as can be seen in Fig. 2d.

**Fig. 3:**
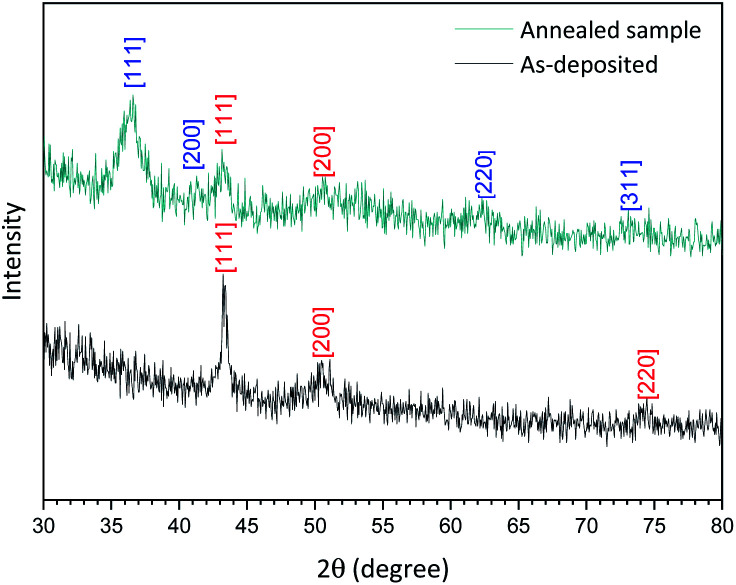
X-ray diffraction spectrum of the as-deposited Cu-NPs (black) and annealed for 20 min at 120 °C (dark cyan) deposited on a silicon substrate. [Numbers] corresponding plane for Cu (red) and Cu_2_O (blue).

The XRD spectrum of the Cu-NPs deposited on a silicon substrate (Fig. 3, ‘as-deposited’ black spectrum) shows peaks at 2*θ* = 43.35°, 50.49° and 74.2° corresponding only to Cu [111], [200] and [220] planes according to the database (JCPDS no 04-0836). The analysis of the crystallite size based on these XRD results was carried out using the Williamson–Hall plot and suggests a mean diameter of 8 nm, which is found to be the same as the one found by TEM analysis (see the ESI S3†). However, the results from TEM analysis are in this case more reliable as XRD analysis generally relies on several parameters (*e.g.* shape factor, strain *etc.*) that cannot be determined accurately and a fitting procedure that cannot always be fully satisfactory.

**Fig. 4:**
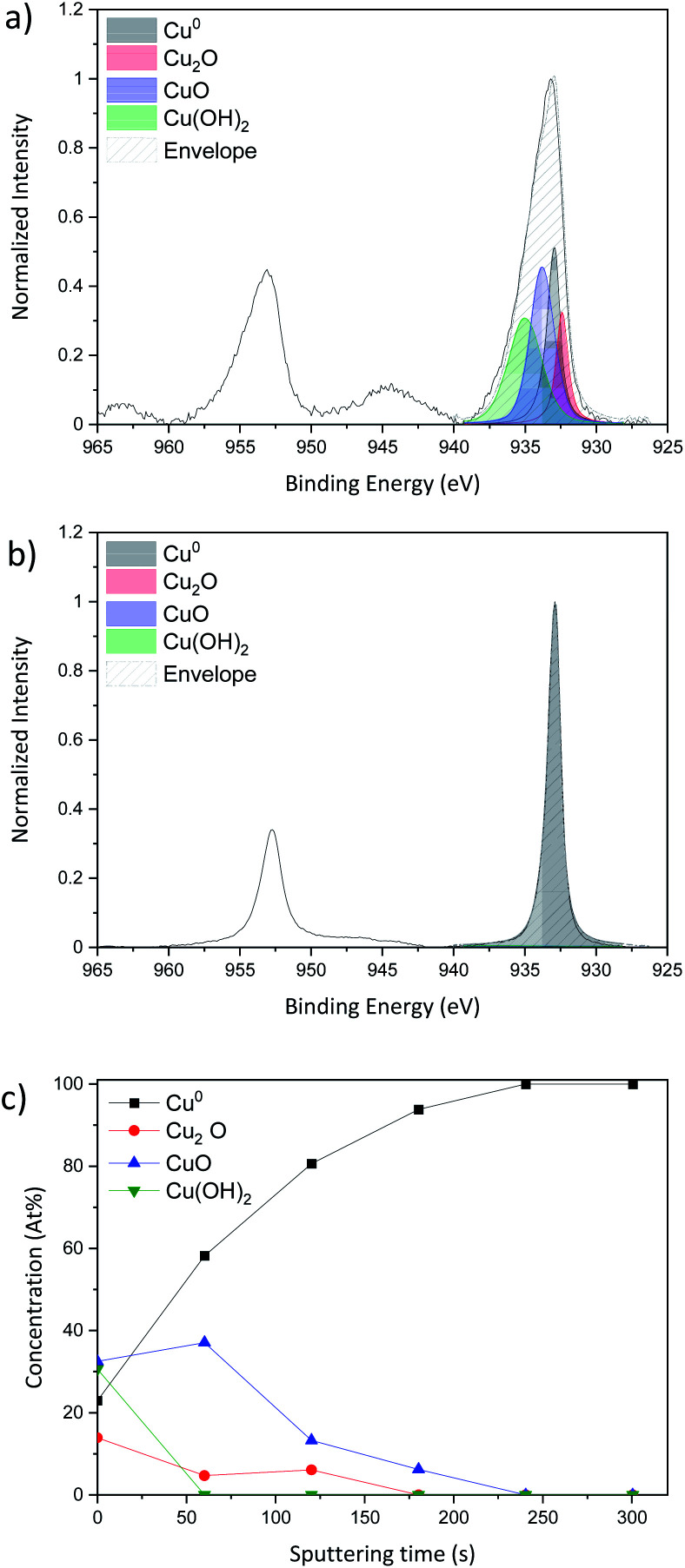
Curve fitting of the high resolution spectra of copper Cu 2p obtained by X-ray photoelectron spectroscopy using depth profile during 300 s; (a) as-deposited sample without sputtering, (b) sample with 30 s Ar^+^ sputtering and (c) evolution of the concentration of the different peaks fitted as a function of the sputtering time of Cu-NPs deposited on a Si substrate.

XPS analysis was carried out in standard analysis mode as well as after Ar^+^ sputtering to produce a chemical depth profile. Depth profiling utilized an argon ion gun at 3 keV for 5 min (300 s) sputtering. Fig. 4a shows the Cu 2p region for the Cu NP as-deposited sample while Fig. 4b reports the spectrum of the same sample after 300 s sputtering, therefore representing the composition below the layer of NPs exposed to air.

The Cu 2p spectra are composed of two main peaks at 932.6 eV and 932.2 eV and these belong respectively to Cu 2p_3/2_ and Cu 2p_1/2_. The Cu 2p_3/2_ only is fitted as the Cu 2p_1/2_ can be fit by applying the spin-orbital split.

According to Biesinger,^43^ the Cu 2p_3/2_ can be fitted with 4 peaks, metallic Cu^0^ at 932.6 eV, copper(i) oxide Cu_2_O at 932.2 eV, copper(ii) oxide CuO at 933.8 eV and finally copper hydroxide Cu(OH)_2_ at 934.7 eV. Even if the XRD spectrum shows only metallic copper diffraction peaks, the XPS shows that the surface of the sample is oxidized as all the species Cu^0^, Cu_2_O, CuO and Cu(OH)_2_ are present. In order to probe deeper into the sample, Ar^+^ sputtering was applied. The result of the Cu 2p spectrum after 300 s of etching and the evolution of each peak during each sputtering step are presented in Fig. 4b and c, respectively.

As the sputtering time progresses, the sample tends to be less and less oxidized giving rise to a pure metallic peak after 300 s of sputtering (Fig. 4b).

As a comparison, we have deliberately oxidized one of our Cu-NP samples by heating in a furnace at 120 °C in air for 15 min and carried out again XRD and XPS analysis.

As shown in Fig. 3, (annealed sample dark cyan spectrum), the XRD spectrum now presents different peaks that can be attributed to a mixture of Cu with planes [111] and [200] and Cu_2_O with planes [111] and [220] confirming that our as-synthesized Cu NPs undergo very limited oxidation. Furthermore, this result corroborates with our XPS depth profile (see the ESI S1†).

The materials characterization therefore shows that metallic Cu-NPs are synthesized with no sign of oxidation, confirmed by our TEM and XRD analysis and corroborated by our XPS depth profiling.

Some of the Cu-NPs however may have undergone oxidation, which is limited to NPs on top of the deposited NP film, whereas buried NPs remain oxide-free. Based on our annealing experiments we can also infer that oxidation of these Cu NPs is relatively slow under ambient conditions. Annealing experiments confirm that oxidation evolves from the NPs exposed to the atmosphere and can reach deeply buried NPs. As confirmed by XRD measurements, after mild annealing, extensive oxidation has taken place.

### CNT/Cu-NP composites

**Fig. 5:**
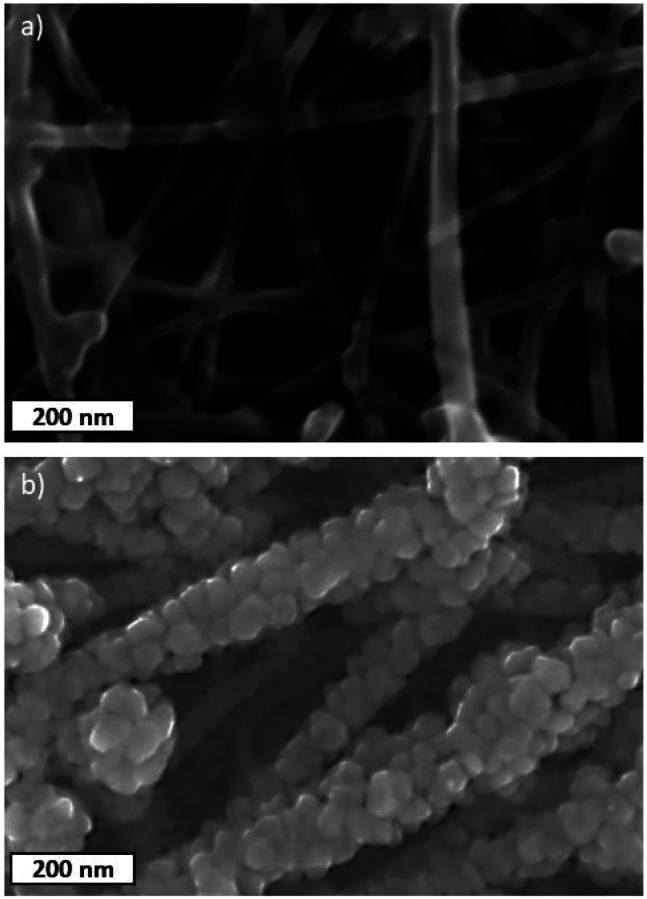
SEM images of (a) pristine CNT ribbons and (b) decorated CNT/Cu-NPs.

The SEM images of the pristine CNT ribbon and CNTs/Cu-NP samples are shown in Fig. 5a and b, respectively. The images clearly show that each multi-wall tube is surrounded by Cu-NPs where the NPs appear agglomerated to form what appears to be 20–40 nm large assemblies. The diameter of the pristine multi-wall CNT lies between 30 nm and 50 nm, which increases to 100–130 nm including Cu-NP deposition.

**Fig. 6:**
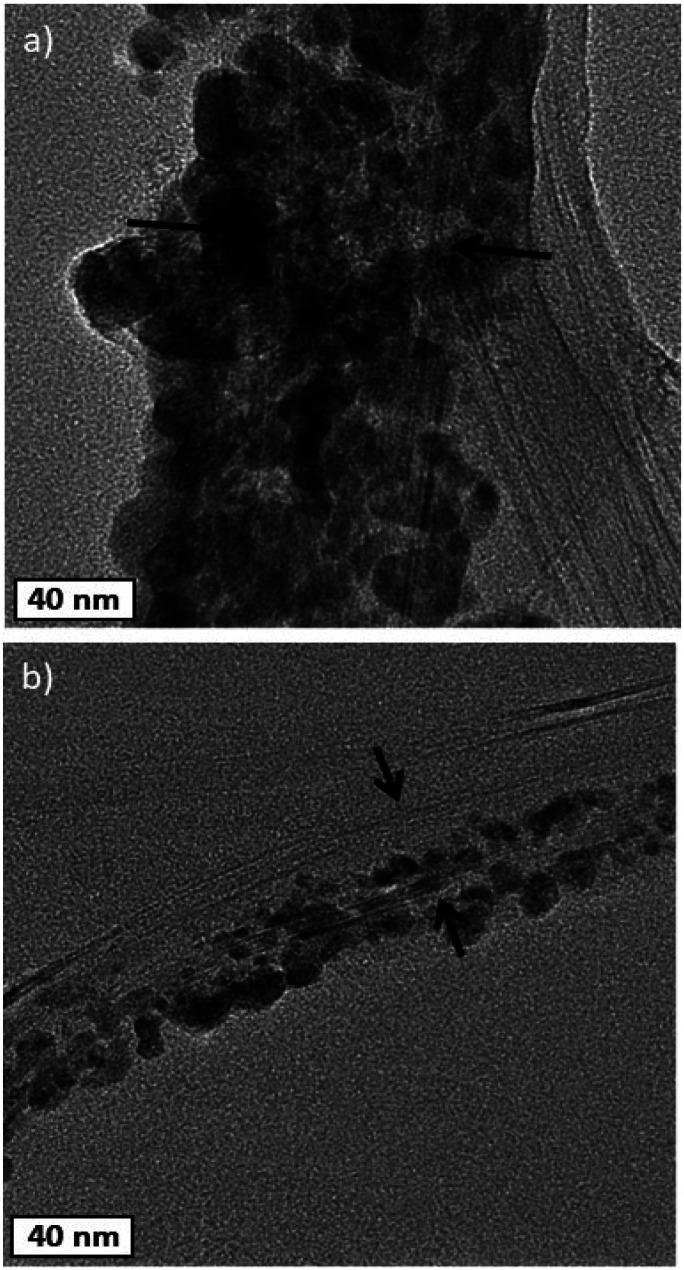
TEM images of decorated CNT/Cu-NPs.

TEM images in Fig. 6a show the Cu-NPs surrounding the CNTs. The multiple walls of the CNT are also clearly visible in Fig. 6b suggesting that the CNT structure is not affected by the Cu-NP deposition process.

In order to provide more details with regard to the composite samples, XPS of pristine CNT ribbons and CNTs/Cu-NP samples was carried out.

The survey spectrum of the pristine CNT ribbon is mainly composed of carbon C 1s with very minor peaks related to oxygen O 1s, iron Fe 2p and sulphur S 2p, the first originating from atmospheric contamination and the other from the catalyst used for CNT growth (see the ESI S2–S4†).

The survey spectrum of the CNTs/Cu-NP composite shows the additional peaks of copper Cu 2p and silicon Si 2p, the first originating from the deposited Cu-NPs and the second from a by-product of the Cu-NP deposition process (see the ESI S5†). More specifically, silicon comes from the use of the quartz capillary (SiO_2_) during the plasma process and we have been able to eliminate completely silicon contamination using an alumina capillary (see the ESI S6†).

**Fig. 7:**
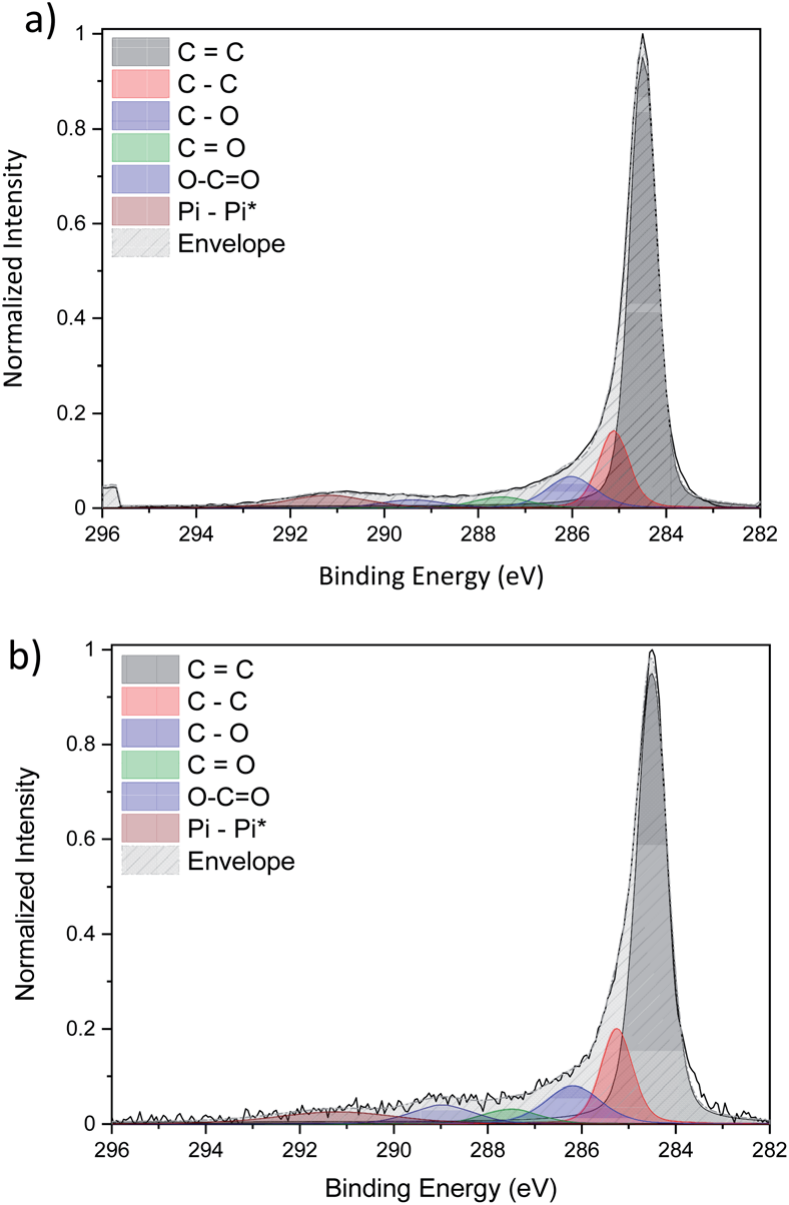
Comparison of the high-resolution C 1s XPS spectra of (a) pristine CNTs and (b) CNT/Cu-NP composite.

The detailed fitting of the high-resolution carbon C 1s peak for both CNT ribbons with (Fig. 7b) and without (Fig. 7a) Cu-NPs present two main peaks attributed to carbon–carbon interaction at 284.5 eV and 284.9 eV which are attributed to sp^2^ and sp^3^ respectively. These two peaks give information on the graphitization of our CNT with a pure sp^2^ carbon corresponding to graphene while a pure sp^3^ carbon belongs to diamond. Three different peaks resulting from the interaction of carbon with oxygen such as C–O, C=O and –COO are also observed at a binding energy of 286 eV, 287.5 eV and 289 eV respectively.

Finally, the peak above 290 eV is associated with π–π* bonds. It is important to note that the fitted C 1s peaks of the pristine CNT ribbon (Fig. 7a) and that of the CNT ribbon with Cu-NPs (Fig. 7b) are essentially the same in terms of peak shape and composition, leading to the conclusion that the Cu-NP deposition does not affect the CNT structure (see the ESI S7†).

**Fig. 8:**
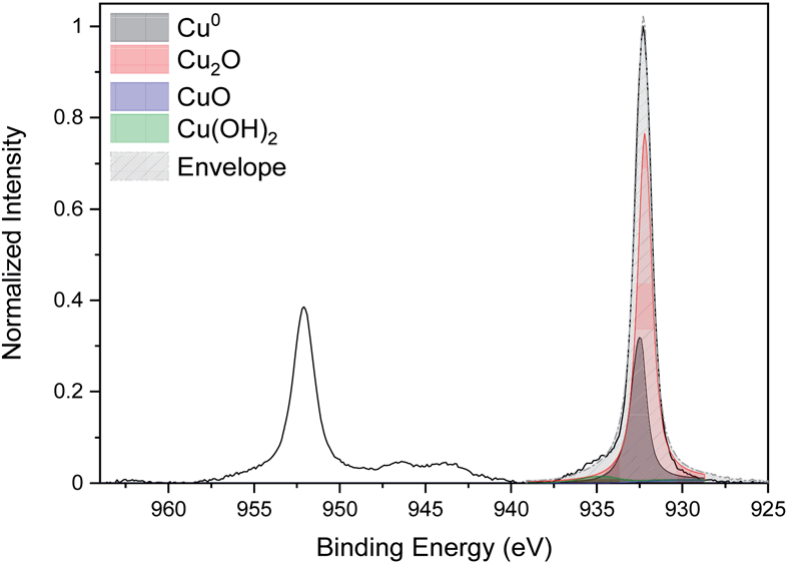
High-resolution XPS spectra of Cu 2p of the CNT/Cu-NP composite.

The curve fitting of the high-resolution spectra of Cu for the CNTs/Cu-NP composite sample is given in Fig. 8. The same fitting procedure as the one followed for Cu-NPs alone (Fig. 4a) was used.

This shows a peak of metallic copper at 932.6 eV but also evidence of oxidation with a strong Cu_2_O peak at 932.2 eV and a Cu(OH)_2_ peak at 934.7 eV.^44^ The oxidation of the Cu NPs on the CNTs is consistent with our analysis of Cu NPs alone; however, the Cu NPs deposited on the CNTs show a higher degree of oxidation. This could be due to the fact that Cu-NPs on CNTs are distributed over a larger surface area and therefore with a larger number of NPs exposed to the atmosphere while Cu-NPs that were deposited separately are for the most part buried in the film with only the top layer exposed to air.

Our analysis of the CNTs/Cu-NP samples demonstrates that CNTs can be successfully decorated with Cu-NPs. CNTs exhibit a high degree of surface coverage and Cu-NPs remain attached to the CNT surface even after sonication (used for TEM analysis), therefore withstanding forces that are able to separate CNTs from each other. However, with these results we cannot yet assess how deep the Cu-NPs have penetrated in the CNT porous structure. The oxidation of the Cu NPs on the CNTs is consistent with our analysis of Cu NPs alone, where the large surface area of the CNTs may have contributed to expose a larger number of NPs to the atmosphere and therefore resulting in a higher degree of oxidation.

## Further characterization of the CNT/Cu-NP composite samples

Important parameters for application of CNT-based composites are related to the amount of NPs included in the CNT support in terms of weight and thickness as well as surface coverage. The weight of the Cu-NPs deposited on the CNTs was therefore evaluated by measuring Cu-NP deposition onto a Si substrate with the same deposition parameters used for deposition on the CNT ribbons; measurements were repeated three times and averaged. This measurement indicated that on average 9 μg of Cu-NPs are deposited, therefore an estimated weight% of 10%. At the same time the sacrificial Cu wire lost on average 90 μg during deposition, indicating that only ∼10% of the Cu from the wire is collected as NPs at the substrate but also providing an upper limit of the deposition potential with this technique.

Porosity and morphology of the CNT ribbons as well as possible large variations in the Cu-NP surface coverage among CNTs, depending on their position within the ribbon, make it quite complicated to produce reliable and relevant figures of merit that are also practical for estimating the thickness of the Cu-NP layer and the CNT surface coverage.^45^

We have therefore tried to capture these characteristics through a semi-quantitative energy dispersive X-ray spectroscopy (EDX) analysis. EDX analysis as a function of the electron accelerating voltage can provide quantitative details on the elemental composition of a sample at various depths.^46^ According to Castaing's approximation,^47^ the penetration depth *H* of an electron used to produce an X-ray depends on the accelerating voltage *E*_0_, the minimum acceleration voltage *E*_c_ which is defined by the energy needed to probe the desired level (electron shell probed) and the intrinsic properties of the element probed (atomic mass *A*, atomic number *Z* and density *ρ*), as in the following eqn (1):1
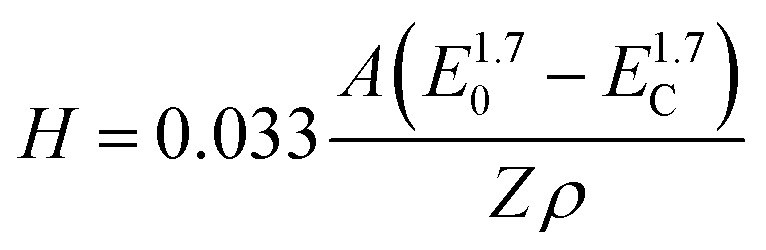


In order to validate this approach, we first used this to estimate the thickness of our CNT ribbons (see the ESI S9†), which indicated a thickness of 7 μm in accordance with our SEM measurements of the CNT ribbon thickness.

**Fig. 9:**
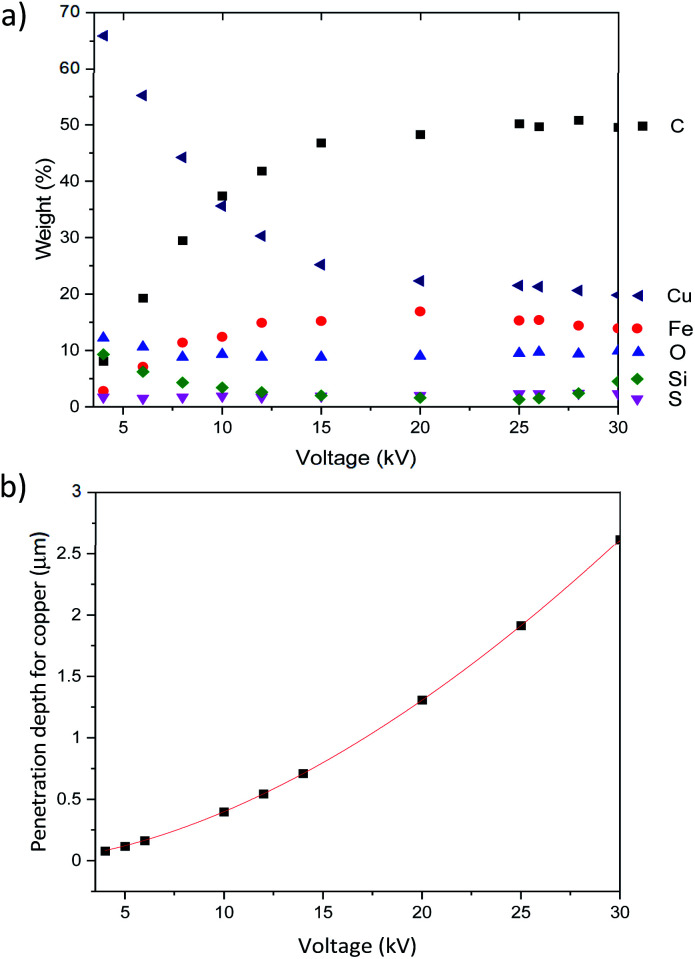
(a) Weight percentage detected by EDX as a function of the accelerating voltage for pristine CNTs and (b) penetration depth through carbon as a function of the accelerating voltage according to Castaings's formula.

We then used this EDX analysis to evaluate Cu-NP thickness at the surface of the CNT ribbons and how deep in the sample Cu NPs could be found. Fig. 9a shows the relative elemental composition as a function of the accelerating voltage for the CNTs/Cu-NP samples. We can generally note two regions, below and above ∼12 kV.

These results show that CNTs can be decorated with Cu-NPs at relatively high weight% (>10%) and depth (∼500 nm) for given applications (*e.g.* in electrochemistry). However we should also note that these figures of merit may be relatively low for other applications where, for instance, an increase in specific conductivity is necessary. Our results, for instance, show that these CNT/Cu-NP composites show very small improvements in the conductivity compared to the CNT ribbon alone (see S8 in the ESI†). This suggests that there may be intrinsic limitations to high-conductivity light-weight composites made of CNTs and copper.

## Conclusion

Cu-NPs have been successfully synthesized using an atmospheric pressure RF microplasma and deposited on a CNT porous structure. Materials characterization and analysis were carried out to confirm the chemical and structural composition of both the nanoparticles and the CNTs/Cu-NP assemblies.

The plasma technique produced metallic Cu-NPs with a mean diameter of ∼8 nm, which underwent oxidation when exposed to the atmosphere. Oxidation can therefore be avoided at the interface between the Cu-NPs and the CNT surface. Analysis showed that the Cu-NP deposition produced very good surface coverage of the CNTs both at the surface of the ribbons and at some depth of up to 500 nm in the porous structure. These results are useful to assess the potential of these composites for relevant applications.

## Conflicts of interest

There are no conflicts to declare.

## Supplementary Material

NA-003-D0NA00922A-s001
