# Supplementary material for: Surfactant-free synthesis of copper nanoparticles and gas phase integration in CNT-composite materials
Source: Nanoscale Adv. 2020 Dec 10;3(3):781–8. doi: 10.1039/d0na00922a (PMC9419625; doi:10.1039/d0na00922a)
Supplement: NA-003-D0NA00922A-s001 [file NA-003-D0NA00922A-s001.pdf]

Electronic Supplementary Material (ESI) for Nanoscale Advances.
This journal is © The Royal Society of Chemistry 2021

Updated: Wednesday 3rd of November, first published 10th of December 2020. This version of the Electronic Supplementary Information replaces a previous copy in where the information on the plasma reactor description and procedures was missing.

Supplementary information S0: Plasma reactor description and procedures

The plasma reactor consists of a quartz capillary (50 mm long) with an internal and external diameter of 0.75 mm and 1.00 mm, respectively. Grounded (top) and radio-frequency (RF) powered (bottom) electrodes with the thickness of 1 mm each spaced by a gap of 2 mm were held fixed around the capillary by a Perspex frame. A copper wire (0.125 mm diameter, Goodfellow Ltd.) is fixed inside the capillary with its end aligned to the powered electrode. An RFPP 500W RF power supply (RF5S) connected to an ENI automatic matching unit (MWH-5-01M9) were used to apply the 13.56 MHz power set at 60 W. The plasma reactor was housed in a stainless-steel chamber, which was pumped down and flushed with a 3 sLm controlled nitrogen to reach again atmospheric pressure. Once atmospheric pressure was reached, this flow was maintained while helium at 0.5 sLm with a mixture of 2% hydrogen in argon at 0.1 sLm were sent through the capillary. The pumping was adjusted to maintain atmospheric pressure. In order to produce a set of reliable and comparable results, the deposition time of Cu NPs was fixed at 20 min and the capillary and the wire were changed after each20 min deposition.

Supplementary information S1: XPS of the annealed Cu-NPs

The curve fitting of the annealed sample before and after 5 min Ar sputtering as well as the evolution of peak fitting during sputtering time are shown in Fig S1a, Fig S1b and S1c respectively.

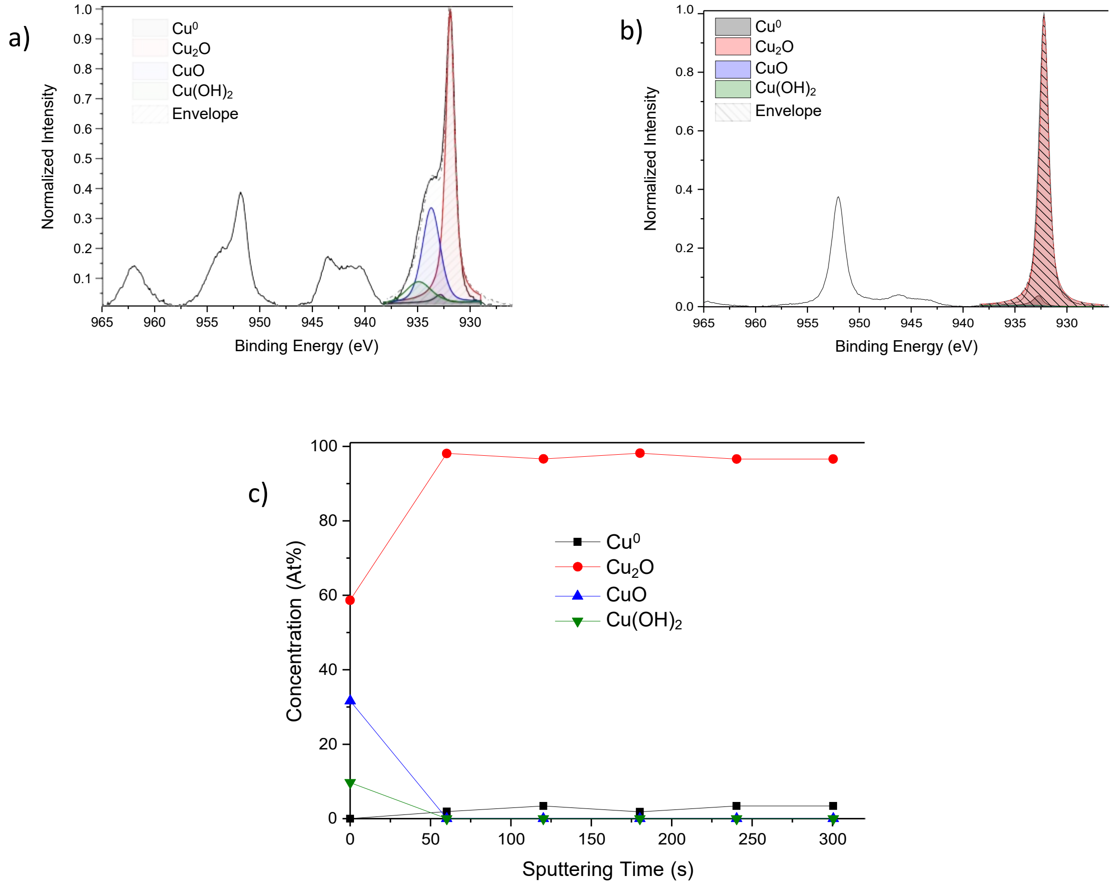

Figure S1: Curve fitting of the high resolution spectra of copper Cu 2p obtained by X-ray photoelectron spectroscopy using depth profile for 300s: a) as-deposited sample without sputtering, b) sample with 300 s sputtering and c) evolution of the concentration of the different peak fitted as a function of the sputtering time of Cu-NPs deposited on Si substrate and anneal in air at 120 °C.

Compared to the as-deposited sample of the Fig 4a, a difference is clearly shown. The peak of Cu $2p_{3/2}$ is broader after annealing and a strong shake-up satellites peak appears between 938 eV and 945 eV. These strong satellites peak refers to hole states in the Cu 3d band of the CuO (Cu $3d^9$ configuration) and are not present in the case of Cu_2O as the 3d band is filled (Cu $3d^{10}$ configuration) and the 4s is unoccupied. However, the presence of the shake-up satellites peak does not exclude the presence of Cu_2O. As it is the case on the curves fitting, where the composition of the anneal sample without sputtering is principally composed of Cu_2O and CuO. After 5 min sputtering, the CuO satellites peaks disappear, leading to a strong reduction of the CuO and an increase in Cu_2O as shown by Fig S1c. The peak related to Cu_2O is predominant after only one sputtering cycle showing that within the depth probed by the combined XPS and sputtering analysis (~10 nm) particles are fully oxidized as only very low quantity of Cu^0 is detected.

Supplementary information S2: Carbon nanotube (CNT) synthesis

The carbon nanotube ribbons in this work are produced in a floating-catalyst chemical vapour deposition system. The iron catalyst nanoparticles are formed by the thermal decomposition of ferrocene powder carried in a carrier gas of hydrogen. Sulphur is a critical precursor for this reaction, therefore thiophene is transported in the carrier gas stream through the furnace. The sulphur fulfils three roles; (1) sulphur acts to enhance the rate of adding carbon to the graphitic lattices, by decreasing the solubility of carbon into liquid iron.[1] As such the carbon atoms must diffuse around the surface of the molten iron catalyst particles in order to nucleate the CNTs.[1–3] (2) Slows down the nucleation barrier for the Fe particle formation – i.e. it influences the aerosol chemistry which is happening, as well as the catalyst-surface effects (Formation of FeS-Fe eutectic island zone or the eutectic causing full surface liquefaction).[2,4,5] (3) Sulphur can prevent the complete encapsulation of the catalyst with amorphous carbons.[6] The hydrogen carrier gas also helps to maintain the catalytic activity of the iron particles by etching amorphous carbon coatings. After several minutes of flowing the ferrocene and thiophene, hydrocarbons are added to the furnace in the form of methane.[7] After the hot zone of the furnace, the material begins to cool and a dense web of dark carbon material forms in the centre of the work tube. This aerogel is sufficiently strong that it can be drawn out with a rod to form a long black "sock".

In order to produce the CNT ribbons in this work, gas flows of 130 standard cubic centimetres per minute (sccm) of hydrogen through the ferrocene bubbler, 90 sccm of hydrogen through the thiophene bubbler, a methane flow rate of 160 sccm and a carrier hydrogen flow rate of 1350 sccm are flown through a tube furnace held at 1290 °C. In the context of this work, the term "ribbon" is used to describe the material obtained by pressing the macroscopic CNT aerogel between two glass microscope slides for 10 minutes under a 2 kg to create a flat ribbon-like material.

(1) Motta, M. S.; Moisala, A.; Kinloch, I. A.; Windle, A. H. The Role of Sulphur in the Synthesis of Carbon Nanotubes by Chemical Vapour Deposition at High Temperatures. *J. Nanosci. Nanotechnol.* **2008**, *8* (5), 2442–2449.

(2) Tibbetts, G. G.; Bernardo, C. A.; Gorkiewicz, D. W.; Alig, R. L. Role of Sulfur in the Production of Carbon Fibers in the Vapor Phase. *Carbon N. Y.* **1994**, *32* (4), 569–576.
(3) Conroy, D.; Moisala, A.; Cardoso, S.; Windle, A.; Davidson, J. Carbon Nanotube Reactor: Ferrocene Decomposition, Iron Particle Growth, Nanotube Aggregation and Scale-Up. *Chem. Eng. Sci.* **2010**, *65* (10), 2965–2977.
(4) Ren, W. et al. The effect of sulfur on the structure of carbon nanotubes produced by a floating catalyst method. J. Nanosci. Nanotechnol.6, 1339–1345 (2006)
(5) Hoecker, C.; Smail, F.; Pick, M.; Weller, L.; Boies, A. M., The Dependence of CNT Aerogel Synthesis on Sulfur-driven Catalyst Nucleation Processes and a Critical Catalyst Particle Mass Concentration. *Scientific Reports* **2017,** *7* (1), 14519
(6) Gspann, T. S.; Smail, F. R.; Windle, A. H. Spinning of Carbon Nanotube Fibres Using the Floating Catalyst High Temperature Route: Purity Issues and the Critical Role of Sulphur. *Faraday Discuss.* **2014**, *173*, 47–65.
(7) Sundaram, R. M.; Koziol, K. K. K.; Windle, A. H. Continuous Direct Spinning of Fibers of Single-Walled Carbon Nanotubes with Metallic Chirality. *Adv. Mater.* **2011**, *23* (43), 5064–5068.

Supplementary Information S2.2: Visual aspect of the CNT

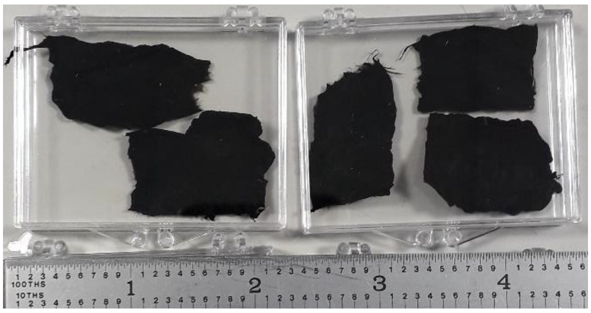

Figure S2.2: Visual aspect of the CNT cut at the desired dimension

Supplementary Information S2.3: SEM images of pristine CNT:

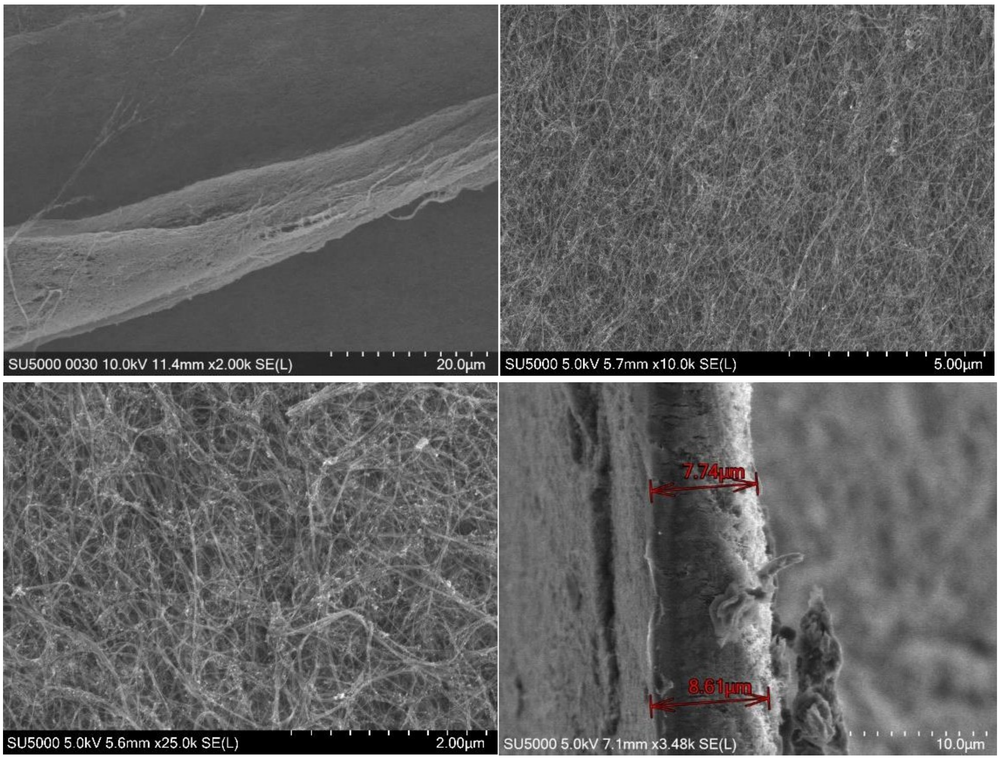

Figure S2.2: SEM image at different magnification x2k, 10k and 25k and thickness of the CNT

SEM images at different magnification show bundle of CNT crossed linked all together. The thickness of the CNT can range between ~3 µm and ~8µm, depending also where the measurement is taken along the ribbon.

<u>Supplementary Information S2.4:</u> TEM images of pristine CNT:

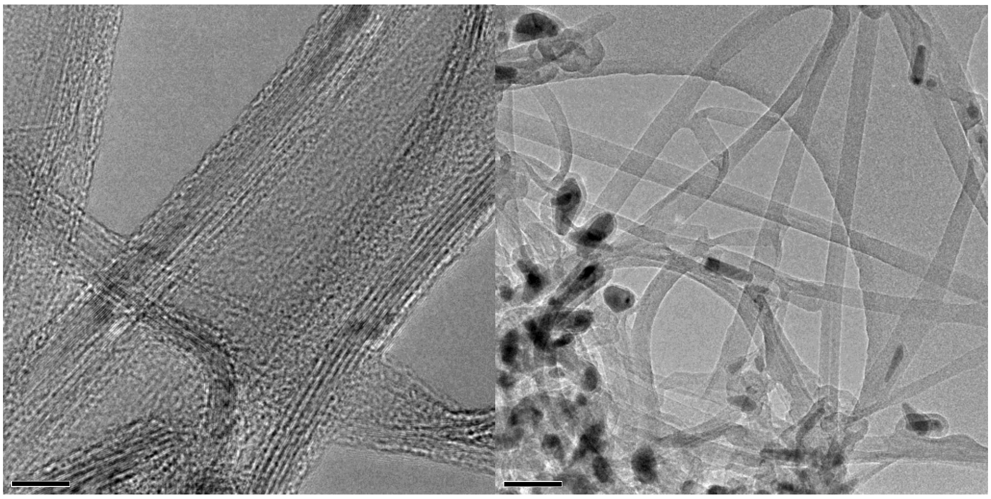

Figure S2.3: HRTEM of pristine CNT and low resolution

The TEM images present the pristine multi wall CNT, as show on the left images. On the right side, we can distinguish the iron particles on which the CNT grow, as well as the CNT themselves. The diameter of the CNT in that case on the range 40-70 nm.

<u>Supplementary information S3</u>: XRD of particles

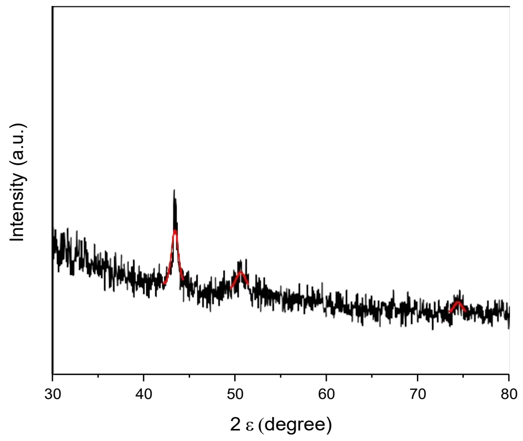

Figure S3.1: XRD of Cu-NPs particles

FWHM (deg)	FWHM (rad) β	2 Θ (deg)	Θ (deg)	Θ (rad)	Cos (Θ)	Sin (Θ)	β*cos(Θ)
0.777	0.01356	43.31	21.655	0.37795	0.92942	0.36902	0.0126
1.06	0.0185	50.52	25.26	0.44087	0.90438	0.42673	0.01673
0.89	0.01553	74.28	37.14	0.64822	0.79716	0.60376	0.01238

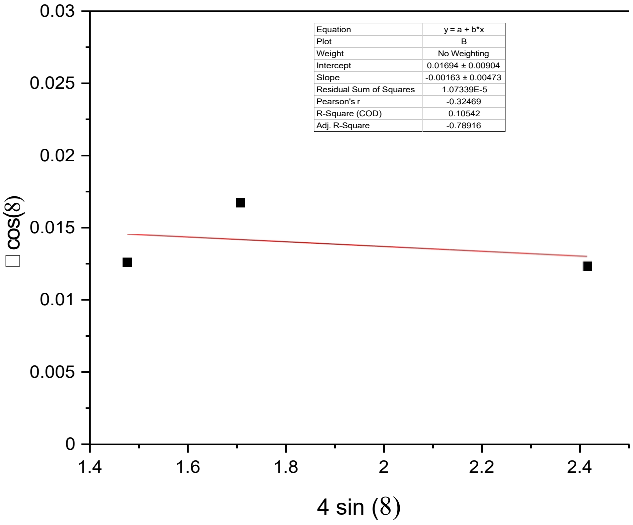

D = (k*λ)/Intercept

k = 0.89

λ = 0.154

intercept = 0.018

Figure S3.2: Williamson-Hall plot of the Cu-NPs

The size of the particles with Williamson-Hall plot was found to be 7.64 nm which is close to the value (~8 nm) found by TEM. However, TEM analysis for size and size distribution remains more reliable. As it can be noticed above, it is difficult to carry out a good fitting of the data points as it is often the case with this type of particles.

<u>Supplementary information S4</u>: XPS of pristine CNT

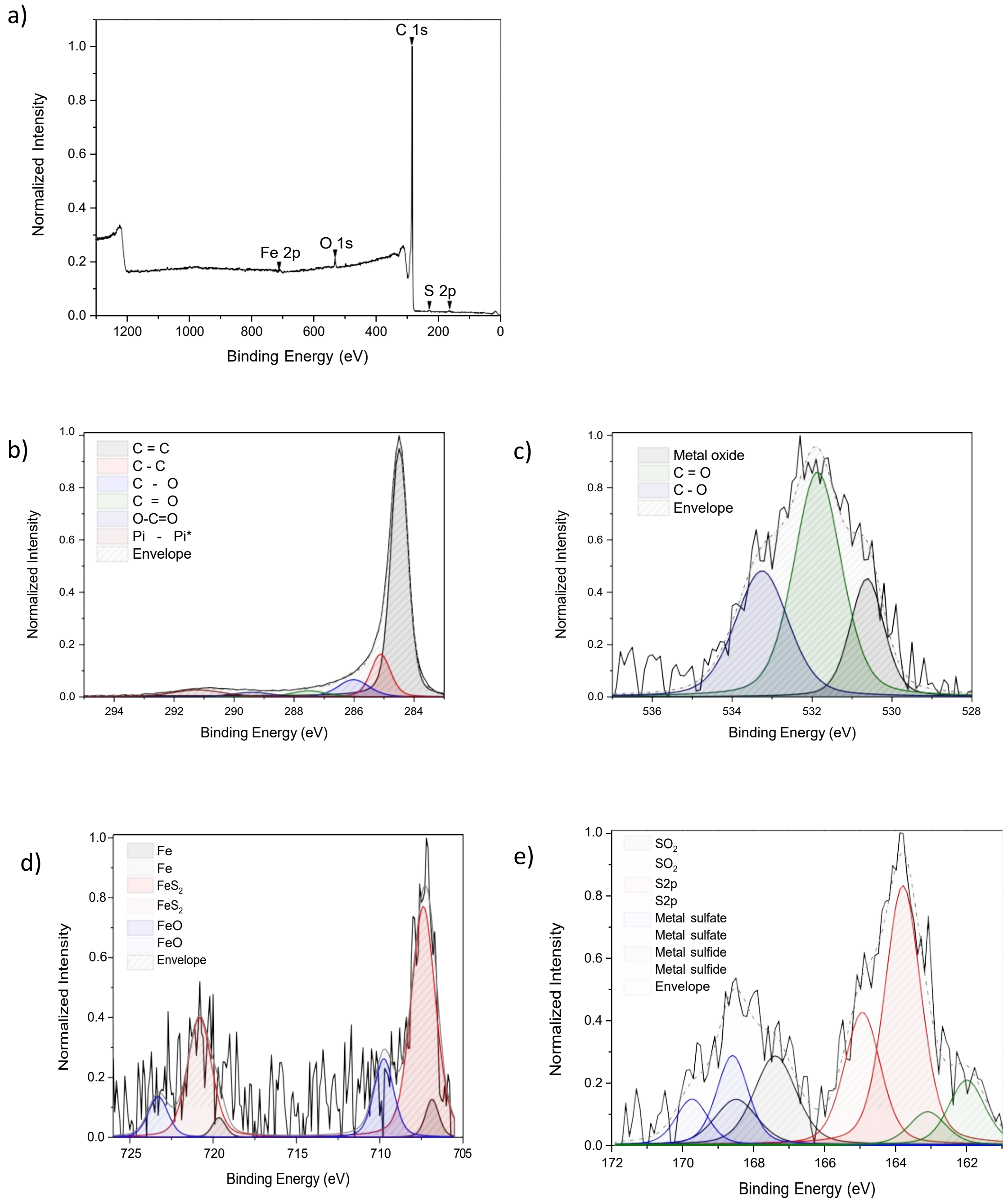

Figure S4: XPS spectrum of pristine CNT a) survey and XPS curve and fitting of high-resolution b) C 1s, c) O 1s, d) Fe 2p and e) S 2p

Supplementary information S5: XPS of composite CNT/Cu-NPs

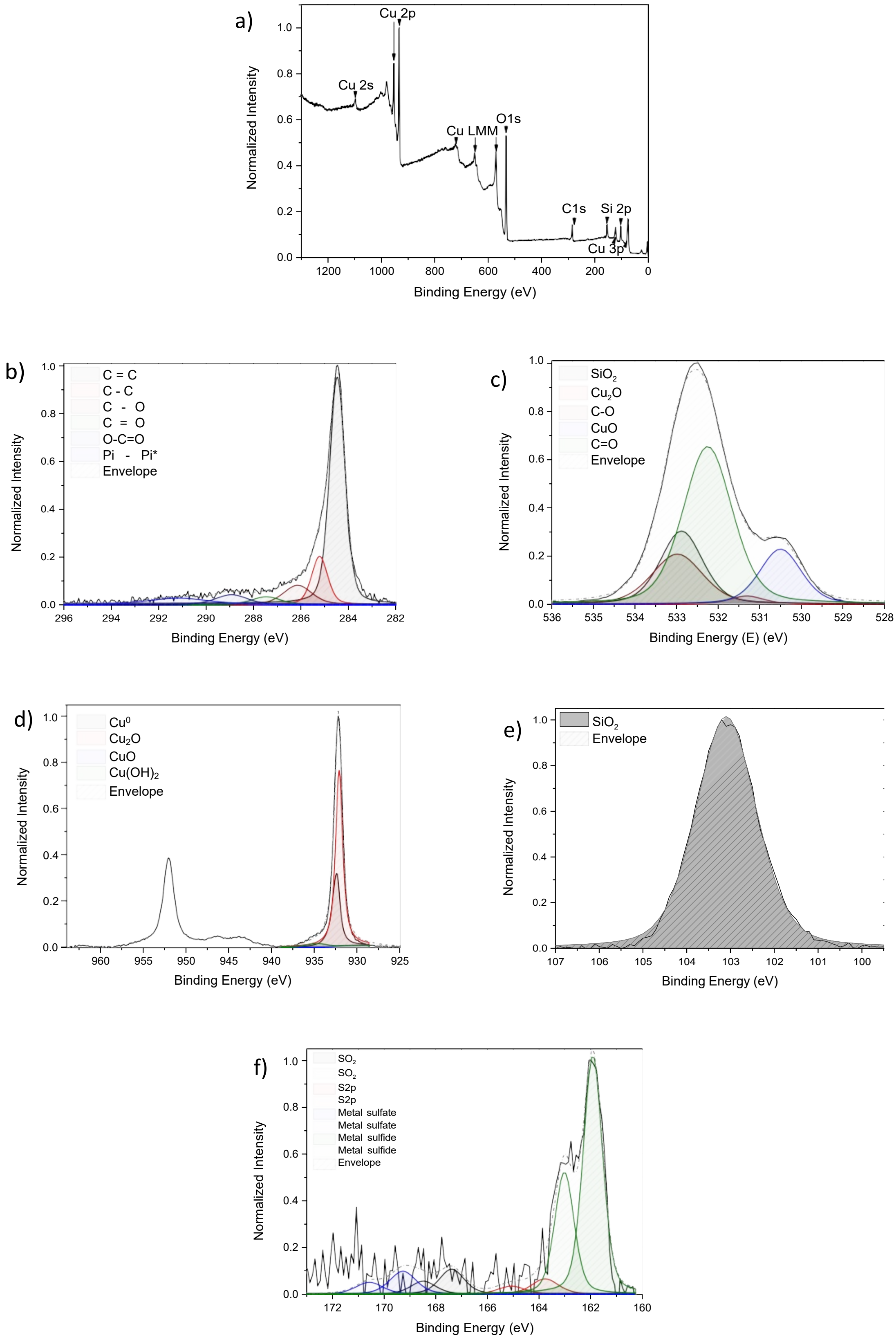

Figure S5: XPS spectrum of the composite CNT/Cu a) survey and XPS curve and fitting of high-resolution b) C 1s, c) Si 2p d) Cu 2p, e) Si 2p and f) S 2p

When comparing between the oxygen spectra O 1s of the pristine CNT Fig 8c and O 1s from the CNT/Cu-NPs Fig 9c, a large increase of the oxygen peak can be observed in the latter. In pristine CNT, the oxygen is present in small quantity and represent defects as the peak at 533.3 eV is related to C-Oand the one 531.8 eV is Cu=O. The other peak at lower binding energy is attributed to metal-oxide dueto presence of iron in our CNT. Once Cu-NPs are deposited, the O 1s peak becomes broader. As Siliconwas found, a peak at 532.9 eV is added to take into account for SiO_2. Also, the presence of Cu_2O at 531.57 eV and CuO at 530.99 eV was added to take surface oxidation into account.

Supplementary information S6: Cu-NPs sample deposited with alumina capillary

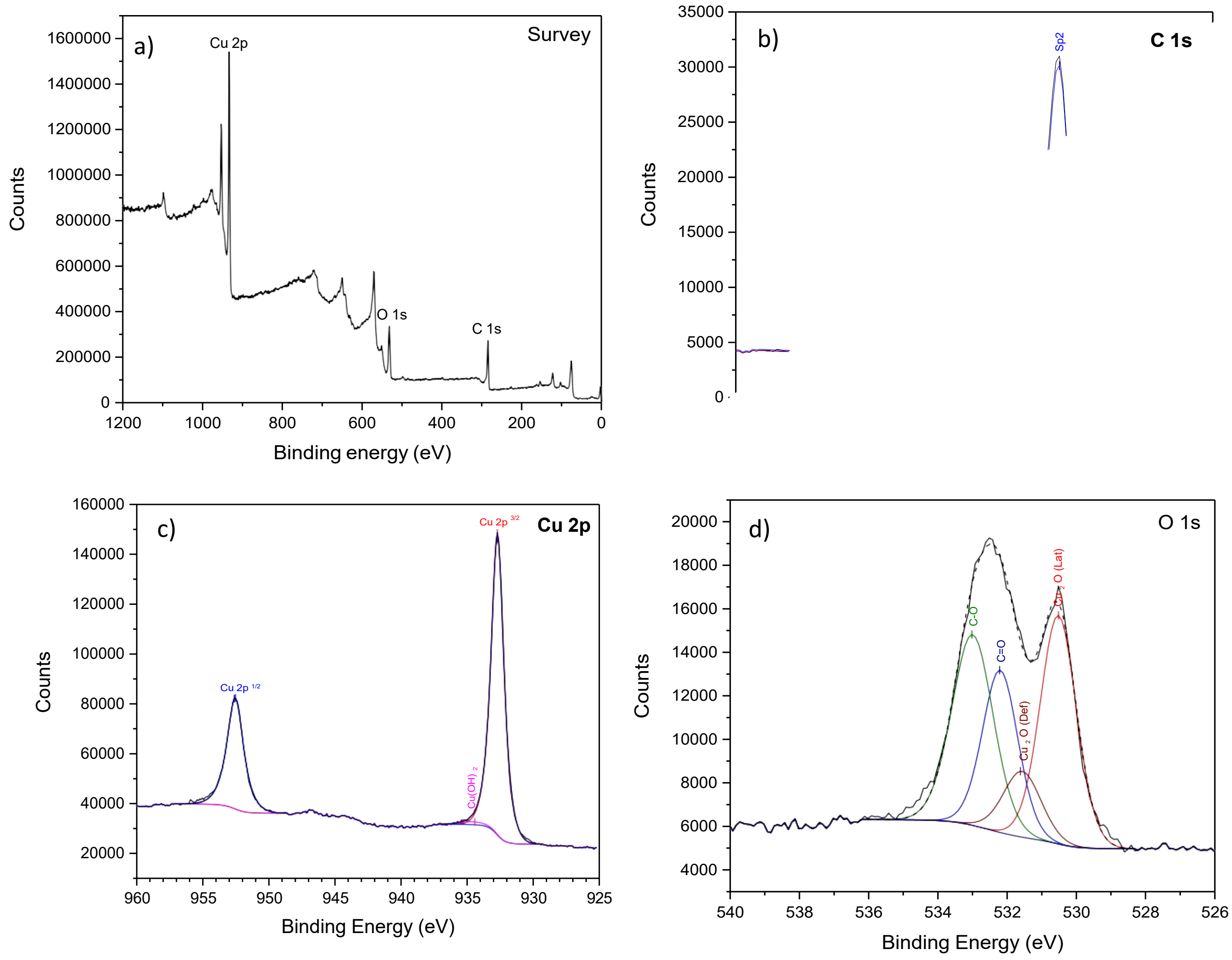

Figure S6: XPS spectrum of the sample deposited with alumina capillary a) survey, b) C 1s, c) Cu 2p and d) O1s

Synthesis of Cu-NPs using an alumina capillary instead of a quartz shows that Si-based contamination can be removed.

Cu-NPs sample deposited with alumina capillary sputtered during 20s

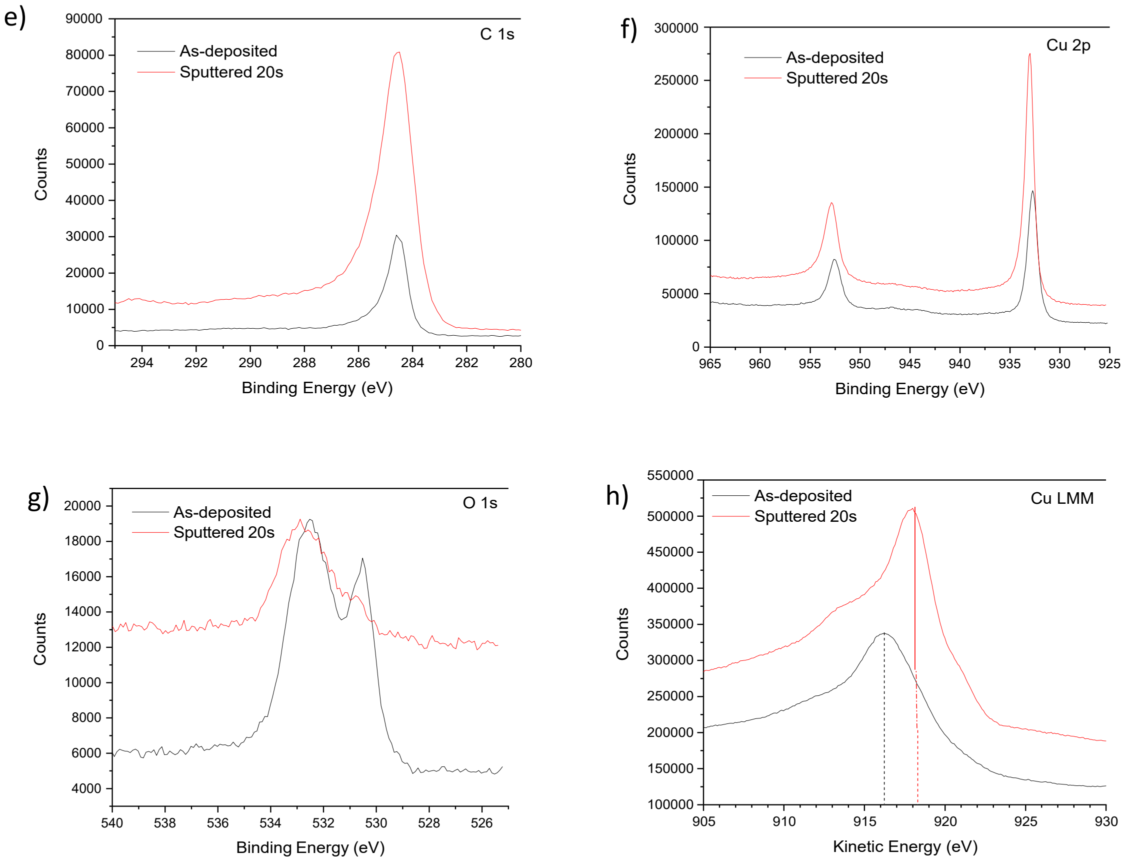

Figure S6: Comparison of the high-resolution XPS spectrum deposited with an alumina capillary and sputtered for 20s for e) Cu 2p, f) Cu LMM, g) O 1s and h) C 1s

As shown above the presence of the oxide on the Cu-NPs can easily be removed using the Ar^+ ions sputtering. As it can be seen after 20 s of sputtering, the Auger LMM peak position shift from 916.8 eV to 918.4 eV confirming the presence of metallic copper. Also, a shift on the main peak Cu 2p is observed and the Cu $2p_{3/2}$ can be fitted with a sharp asymmetric peak as it is the case for metallic copper. At the same time on the O1s spectra, the peak relative to Cu_2O disappear, giving rise to an SiO_2 peak.
A common way to observe the oxidation is by analyzing the auger Cu_{LMM} peak, as the shift on the Auger LMM between Cu and Cu_2O is 1.8 eV. The position of the Auger Cu_{LMM} peak was found to be at 916.8 eV confirming the oxidation Cu_2O more than Cu.

<u>Supplementary information S7</u>: Raman spectra of Pristine CNT and CNT/Cu-NPs

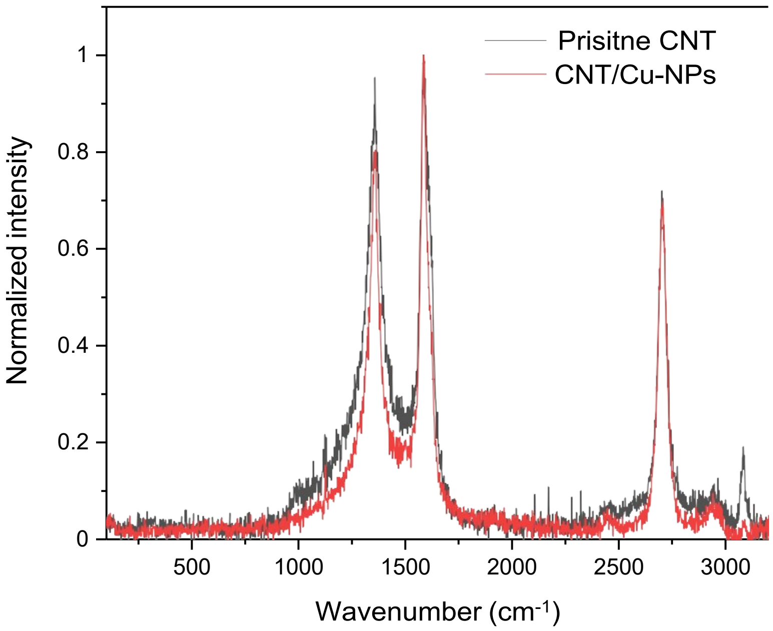

Figure S7: Raman spectra of pristine CNT in black and CNT/Cu-NPs in red

On the Fig. S7 of the pristine CNT we can see the characteristics band of MWCNTs, D band related to defects is shown at 1350 cm^{-1}, the G band related to the presence of graphite is shown at 1580 cm^{-1}, the other band at 2700 cm^{-1} is called G' band and is an overtone of the D band. Once treated, no other band was found. As the metallic copper is not supposed to show any band, CuO or Cu_2O presence is usually in the range of 290-650 cm^{-1}.

Supplementary Information S8: Conductivity of the CNT samples

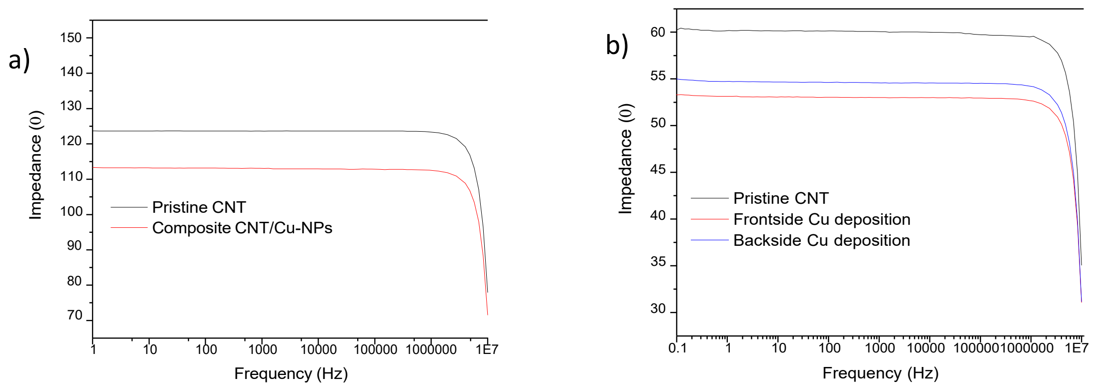

Figure S8: Impedance measurement of the CNT and CNT/Cu-NPs as a function of the frequency for CNT ribbons with Cu-NPs deposited a) on one side only and b) both sides.

In order to compare the electrical conductivity of the pristine CNT to the composite CNT/Cu-NPs sample, a Perspex frame was made to support the CNT during the plasma treatment but also to carry out a four-probe measurement before and after deposition without any manipulation of the CNT in between each step. Also, measurements for the pristine CNT ribbon and for the CNT ribbon exposed to a plasma without Cu-NPs deposition were carried out for comparison. The samples were analyzed using an AC impedance spectroscopy measurement with a Novocontrol 4 wires system. The result shows the evolution of the impedance as a function of the frequency for a single face treated Fig S6a and for both faces treated Fig S6b. This clearly show a decrease of the impedance, and so an increase of the conductivity when the CNT is covered with Cu-NPs. However the improvement is currently limited.

<u>Supplementary information S9</u>: Thickness evaluation with EDX

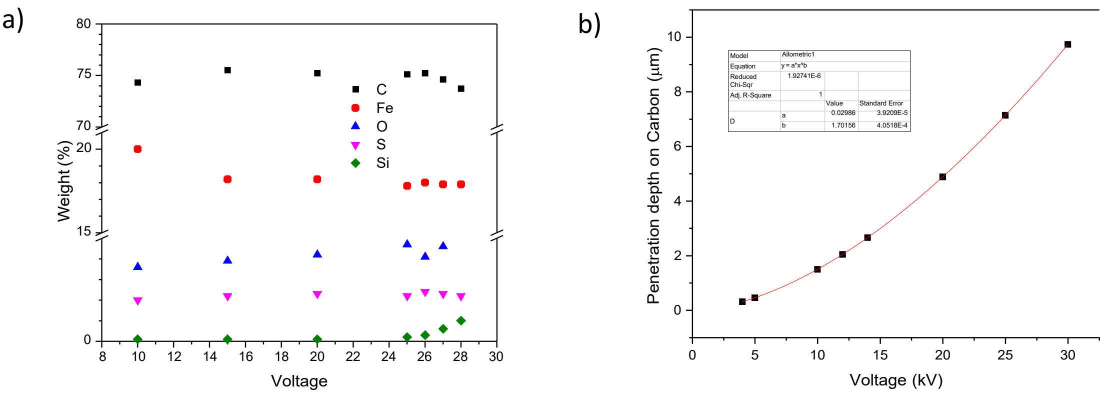

Figure S9: a) Weight percentage detected by EDX as a function of the accelerating voltage for pristine CNT and b) penetration depth through carbon as a function of the accelerating voltage according to Castaing's formula

All the elements present in the CNT were constant independent of the accelerating voltage. The chemical composition of the CNT does not vary across the thickness. It was found that the acceleration voltage needed to start detecting the substrate was slightly higher than 25 kV. This value was reported on the curve shown in Fig.S8b, which represent the penetration depth for the carbon as a function of the accelerating voltage according to the Castaings's formula. If we report the value obtained 25 kV on the Castaings's approximation we obtain a value of 7 µm.
